# Large intramural hematoma of the esophagus after endoscopic injection sclerotherapy: A case report

**DOI:** 10.1097/MD.0000000000032752

**Published:** 2023-01-27

**Authors:** Bo Wu, Xincheng Xie, Xiao Li, Qun Zhu, Chunhua Zhou

**Affiliations:** a Department of Gastroenterology, HangZhou Xixi Hospital, Hangzhou, China.

**Keywords:** endoscopic injection sclerotherapy, esophageal hematoma, esophageal varices

## Abstract

Endoscopic injection sclerotherapy (EIS) is a common treatment for patients with liver cirrhosis and esophageal varices. It can effectively treat variceal rupture and bleeding caused by liver cirrhosis. However, EIS has many complications, including postoperative bleeding, retrosternal pain, esophageal ulcers, esophageal stenosis, and ectopic embolism. Intramural hematoma of the esophagus (IHE) is a rare complication of EIS that can lead to chest tightness, chest pain, and dysphagia.

**Patient’s concerns:**

A 55-year-old man developed severe nausea and vomiting accompanied by chest pain after EIS.

**Diagnosis:**

Comprehensive imaging features, the patient was diagnosed as IHE.

**Interventions:**

A vascular clamp was used for hemostasia, and a feeding tube was placed in the patient’s jejunum.

**Outcomes:**

After the removal of the jejunal feeding tube and the intake of a semiliquid diet, the patient had no episodes of chest pain, chest tightness, or dysphagia and was discharged after 2 days of observation.

**Lessons:**

Although IHE rarely occurs after EIS, we should not overlook its risk. The occurrence of IHE is not directly related to the number of EISs received or the degree of liver cirrhosis but is more likely related to postoperative nausea and vomiting. Therefore, timely medication and observation are particularly important for patients with nausea and vomiting after endoscopic treatment.

## 1. Introduction

Intramural hematoma of the esophagus (IHE) is a rare disease that can be classified into spontaneous and secondary IHE. IHE is caused by submucosal hemorrhage or esophageal mucosal dissection, which is related to vomiting, foreign bodies, endoscopic injury, hemophilia, or oral administration of aspirin.^[1–6]^ The typical symptoms of IHE include dysphagia, chest pain, and hematemesis,^[7–9]^ but these symptoms rarely occur at the same time. IHE with symptoms such as chest tightness, chest pain, and inability to lie down should be distinguished from cardiovascular diseases such as acute myocardial infarction and aortic dissection. computed tomography (CT) and endoscopy are effective methods for the diagnosis of IHE. The endoscopic manifestation can be a mucosal bulge of the esophagus with a dark-blue surface. IHE typically has good prognosis and can spontaneously subside within 3 to 4 weeks.

## 2. Case presentation

**Figure 1.**
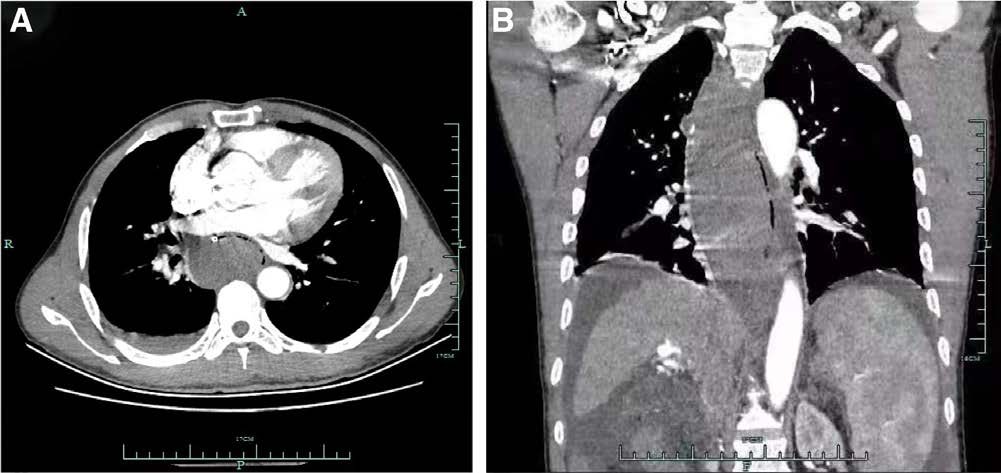
Chest CT images. (A) Axial view showing esophageal stenosis and a soft tissue opacity. (B) Nonenhanced esophageal mass involving the entire esophagus in the mediastinum. CT = computed tomography.

**Figure 2.**
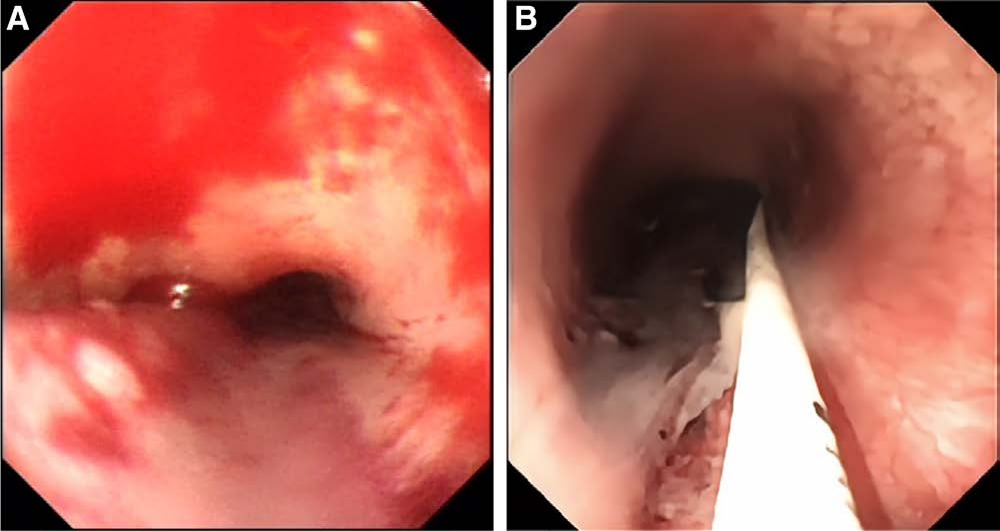
Gastroscopy images. (A) When the gastroscope was advanced forward, a mucosal bulge with a light-blue surface in the lower esophagus was observed. (B) When the gastroscope was retracted, it was observed that as the mucosal bulge extended to the upper esophagus, its width decreased, and its surface became bluish.

**Figure 3.**
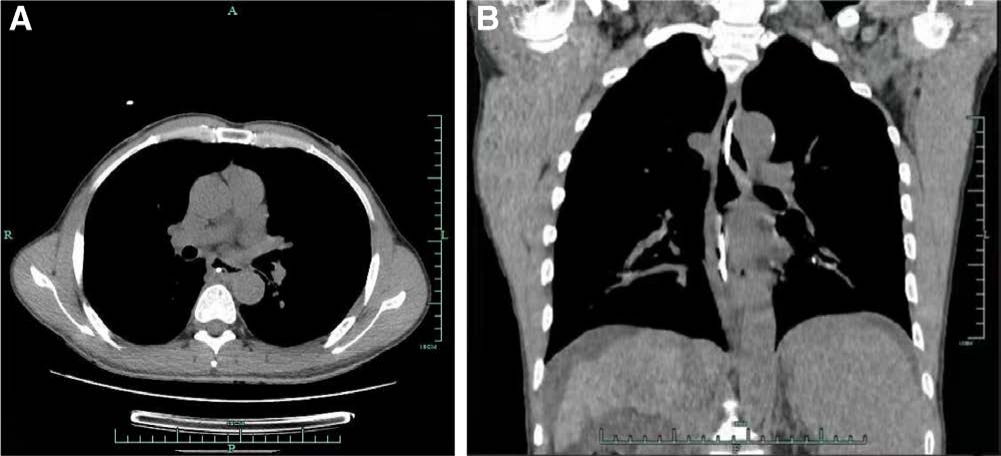
CT images. Axial view (A) and coronal view (B) indicating the disappearance of the esophageal mass and the improvement of esophageal stenosis. CT = computed tomography.

**Figure 4.**
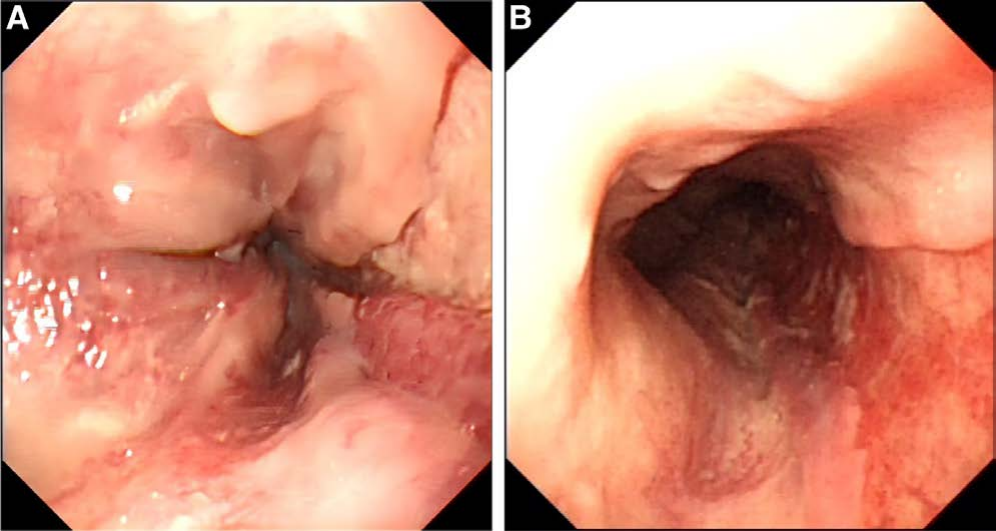
Gastroscopy images. (A) Local mucosal damage in the lower esophagus; (B) exposed submucosal tissues in the middle and upper esophagus.

A 55-years-old man was diagnosed with posthepatitis B cirrhosis for 4 years, for which he underwent endoscopic injection sclerotherapy (EIS) in July 2021 and November 2021. In July 2022, the patient was hospitalized again in our hospital. Upon admission, his platelet count was 56 × 10^9^/L, and his coagulation test results were as follows: prothrombin activity 68.7%, international normalized ratio 1.18, fibrinogen 1.76 g/L, and D-dimer concentration 2.05 mg/L. On July 1, 2022, the patient received EIS with tissue gel and sclerosant. Shortly after EIS, the patient developed severe nausea and vomiting accompanied by chest pain, which were relieved after antiemetic and analgesic treatment. From the night of July 1 to July 2, the patient had a total of 3 melena episodes and complained of persistent and worsening chest pain, as well as dysphagia and chest tightness. The patient had to stay in a semireclined position due to these symptoms. Electrocardiogram suggested tachycardia (heart rate > 100 beat/min). After that, the patient had hematemesis once, and he immediately had the myocardial enzyme spectrum analyzed and underwent another electrocardiogram, which ruled out the possibility of acute coronary syndrome. CT examination indicated an intramural esophageal soft tissue opacity extending longitudinally from the first thoracic layer to the fundus of the stomach, with an elliptical shape on the axial view and a CT value of 50 HU (Fig. 1). Hence, the patient was likely to have a intramural hematoma of the esophagus (IHE). Gastroscopy showed a dark blue mucosal bulge in the middle and lower esophagus (Fig. 2), no obvious bleeding in the esophageal lumen, and a scab at the varix of the fundus of the stomach. A vascular clamp was used for hemostasia, and a feeding tube was placed in his jejunum. Later, the patient was treated with nasal feeding, human albumin supplementation, and oral thrombin, which significantly improved the chest pain symptoms. Ten days after this treatment, CT suggested the disappearance of the hematoma (Fig. 3), and gastroscopy indicated the disappearance of the hematoma in the middle and lower esophagus, locally exposed submucosal tissues, and no discernible old bleeding spots in the whole esophagus (Fig. 4). After the removal of the jejunal feeding tube and the intake of a semiliquid diet, the patient had no episodes of chest pain, chest tightness, or dysphagia and was discharged after 2 days of observation. At the time of the telephone follow-up, the patient felt good and had had no relapse of intramural hematoma of the esophagus (IHE).

## 3. Discussion

Although IHE rarely occurs after EIS, we should not overlook its risk. In particular, IHE should be differentiated from complications such as postoperative bleeding and ectopic embolism after chest pain and vomiting occur.^[10,11]^ In general, the symptoms of IHE appear within a few hours after endoscopic treatment, but the peak of symptoms in this case occurred more than 24 hours after EIS. There have been few reports of IHE secondary to EIS since 1990. Based on this case report and a literature review, we found that IHE did not occur in patients who had received more rounds of EIS or had thrombocytopenia, which seems to indicate that the occurrence of IHE is not directly related to the number of EISs received or the degree of liver cirrhosis but is more likely related to postoperative nausea and vomiting. Therefore, timely medication and observation are particularly important for patients with nausea and vomiting after endoscopic treatment.

## Author contributions

**Data curation:** Qun Zhu, Chunhua Zhou.

Formal analysis: Bo Wu.

Resources: Xiao Li.

Writing – original draft: Bo Wu.

Writing – review & editing: Xincheng Xie.
